# Supplementary material for: MetaQTL: a package of new computational methods for the meta-analysis of QTL mapping experiments
Source: BMC Bioinformatics. 2007 Feb 8;8:49. doi: 10.1186/1471-2105-8-49 (PMC1808479; doi:10.1186/1471-2105-8-49)
Supplement: Additional File 7 — MetaQTL Package : jar file and tutorial. This Zip archive contains both the MetaQTL JAR file and the files of the tutorial. [file 1471-2105-8-49-S7.zip › org.inra.metaqtl/doc/org/metaqtl/TreeNode.html]

TreeNode


|  |  |  |
| --- | --- | --- |
| **Overview** \| **Package** \| **Class** \| **Use** \| **Tree** \| **Deprecated** \| **Index** \| **Help** | |  |
| **PREV CLASS**   NEXT CLASS | **FRAMES**    **NO FRAMES**     **All Classes** |  |
| SUMMARY: NESTED \| FIELD \| CONSTR \| METHOD | DETAIL: FIELD \| CONSTR \| METHOD |  |


---


## org.metaqtl Class TreeNode

```
java.lang.Object
  org.metaqtl.TreeNode
```

**All Implemented Interfaces:**: java.io.Serializable

---

``` public class TreeNode extends java.lang.Object implements java.io.Serializable ```

This class represents a node in a binary Tree obtained
when applying usual hirarchical clustering methods.
An array of `TreeNode` can then be viewed as
a tree.

**See Also:**: Serialized Form

---

| **Field Summary** | |
| --- | --- |
| `int` | `card`             The number of sequences in the cluster |
| `TreeNode[]` | `children`             Left and right children, or null |
| `double` | `dist`             The distance between the node and its parent. |
| `double` | `height`             The distance between the node and the root of the tree (if defined). |
| `int` | `idx`             ? The identifiant of the cluster. |
| `java.lang.String` | `label`             The label of the cluster |
| `boolean` | `leaf`             The status of the node. |
| `int[]` | `nidx`             The indices of the sub-nodes |
| `TreeNode` | `parent`             The parent of the node |
| `double` | `x` |
| `double` | `xc` |
| `double` | `y` |


**Constructor Summary**

`TreeNode()`
  
          Creates a new instance of `TreeNode` with
default attribute values.

`TreeNode(int idx)`
  
          Creates a new instance of `TreeNode`
with the given indice `idx`.

`TreeNode(int idx,
TreeNode[] children)`
  
          Creates a new instance of `TreeNode` with the
given indice `idx` and the children of the node.


| **Method Summary** | |
| --- | --- |
| `void` | `addChild(TreeNode node)`             Add a child to the node. |
| `int` | `getCard()` |
| `double` | `getCentroid(double[] y2, double[] sd)` |
| `TreeNode[]` | `getChildren()` |
| `double` | `getDist()` |
| `double` | `getHeight()` |
| `java.lang.String` | `getName()` |
| `TreeNode` | `getParent()` |
| `double` | `getX()` |
| `double` | `getY()` |
| `void` | `setCentroid(double xc)` |
| `void` | `setX(double x)` |
| `void` | `setY(double y)` |

**Methods inherited from class java.lang.Object**

`clone, equals, finalize, getClass, hashCode, notify, notifyAll, toString, wait, wait, wait`

**Field Detail**

### idx

```
public int idx
```

:   ?
    The identifiant of the cluster.

---


### label

```
public java.lang.String label
```

:   The label of the cluster

---


### card

```
public int card
```

:   The number of sequences in the cluster

---


### nidx

```
public int[] nidx
```

:   The indices of the sub-nodes

---


### leaf

```
public boolean leaf
```

:   The status of the node.

---


### dist

```
public double dist
```

:   The distance between the node and its parent.

---


### height

```
public double height
```

:   The distance between the node and the root of the tree (if defined).

---


### children

```
public TreeNode[] children
```

:   Left and right children, or null

---


### parent

```
public TreeNode parent
```

:   The parent of the node

---


### x

```
public double x
```

---


### y

```
public double y
```

---


### xc

```
public double xc
```


**Constructor Detail**

### TreeNode

```
public TreeNode(int idx)
```

:   Creates a new instance of `TreeNode`
    with the given indice `idx`. Use it
    to create the leaves of the tree.

    **Parameters:**: `idx` - the indice of the node.

---


### TreeNode

```
public TreeNode(int idx,
                TreeNode[] children)
```

:   Creates a new instance of `TreeNode` with the
    given indice `idx` and the children of the node.
    Use this constructor to create the internal nodes of the tree.

---


### TreeNode

```
public TreeNode()
```

:   Creates a new instance of `TreeNode` with
    default attribute values.


**Method Detail**

### addChild

```
public void addChild(TreeNode node)
```

:   Add a child to the node.

    :   **Parameters:**: `node` -

---


### getName

```
public java.lang.String getName()
```

:   **Returns:**

---


### getChildren

```
public TreeNode[] getChildren()
```

:   **Returns:**

---


### getCard

```
public int getCard()
```

:   **Returns:**

---


### getHeight

```
public double getHeight()
```

---


### setX

```
public void setX(double x)
```

:   **Parameters:**: `x` -

---


### setY

```
public void setY(double y)
```

:   **Parameters:**: `x` -

---


### getY

```
public double getY()
```

:   **Returns:**

---


### getX

```
public double getX()
```

---


### getDist

```
public double getDist()
```

:   **Returns:**

---


### getParent

```
public TreeNode getParent()
```

:   **Returns:**

---


### setCentroid

```
public void setCentroid(double xc)
```

:   **Parameters:**: `d` -

---


### getCentroid

```
public double getCentroid(double[] y2,
                          double[] sd)
```

:   **Parameters:**: `y2` -: `sd` -


---


|  |  |  |
| --- | --- | --- |
| **Overview** \| **Package** \| **Class** \| **Use** \| **Tree** \| **Deprecated** \| **Index** \| **Help** | |  |
| **PREV CLASS**   NEXT CLASS | **FRAMES**    **NO FRAMES**     **All Classes** |  |
| SUMMARY: NESTED \| FIELD \| CONSTR \| METHOD | DETAIL: FIELD \| CONSTR \| METHOD |  |


---
